# Ureteroscopic Cryoablation for Patients with Upper Tract Urothelial Carcinoma of a Solitary Kidney: A Porcine Model and Our Pilot Clinical Experience

**DOI:** 10.1245/s10434-021-10233-5

**Published:** 2021-06-15

**Authors:** Lujia Zou, Rongzong Liu, Chenyang Xu, Chen Yang, Zheyu Zhang, Jimeng Hu, Haowen Jiang

**Affiliations:** 1 Department of Urology, Huashan Hospital, Fudan University, Shanghai, People’s Republic of China; 2 Institute of Urology, Fudan University, Shanghai, People’s Republic of China; 3 National Clinical Research Center for Aging and Medicine, Huashan Hospital, Fudan University, Shanghai, People’s Republic of China

## Abstract

**Purpose:**

To investigate the safety and efficacy of ureteroscopic cryoablation by a liquid-nitrogen system in a porcine model and for patients with upper tract urothelial carcinoma (UTUC) of a solitary kidney.

**Methods:**

In the animal experiment, the right-sided ureter was frozen in nine pigs. Eight were randomly assigned to two different groups according to the freezing duration of 60 or 90 s. The other one was designed to receive a 10-min freeze. The treated ureters were harvested at 30 min, 2 days, 4 weeks, and 3 months after cryoablation for histological evaluation. After the animal study, we conducted a pilot clinical trial that enrolled six patients who were diagnosed with UTUC of a solitary kidney and received therapeutic management with ureteroscopic cryoablation at our center. Perioperative adverse events and oncological outcomes were evaluated.

**Results:**

In the porcine model, the liquid-nitrogen system was capable of forming a therapeutic ice ball which infiltrated the full-thickness ureter and induced apoptosis and necrosis from mucosa to lamina muscularis through histological examination. In the clinical trial, cryoablation was successfully performed under ureteroscopy in all the patients, without intraoperative ureteral perforation, avulsion, or active hemorrhage. No recurrence in situ was observed during a median follow-up period of 12.5 months. Hydronephrosis and ureteral stricture was observed in one patient and was managed with ureteroscopic balloon dilation.

**Conclusions:**

Ureteroscopic cryoablation induced by liquid nitrogen is a promising technique for conservative management of UTUC with benefits of improving local tumor control and preservation of a solitary kidney.

**Supplementary Information:**

The online version contains supplementary material available at 10.1245/s10434-021-10233-5.

Radical nephroureterectomy (RNU) with bladder cuff resection is considered as standard treatment for localized high-risk upper tract urothelial carcinoma (UTUC).^1^ Over recent decades, advances in endoscopic technique have promoted the development of management for UTUC. For low-risk UTUC, the oncological outcomes of kidney-sparing surgery (KSS) are comparable to RNU, without the complications associated with radical surgery^2^ In patients with high-risk UTUC, KSS can also be considered in some imperative indications, such as solitary kidney, bilateral UTUC, or renal insufficiency. However, conventional endoscopic management through ureteroscope or percutaneous access for low-risk UTUC is associated with increased risk of recurrence and progression^3^^,^ 4 and consequently requires improvement.

Cryoablation offers the potential advantage of treating full-thickness urinary tract wall through ice ball^5^ Several previous studies have validated the safety and efficacy of applying cryotherapy in bladder or for bladder tumor through percutaneous or transurethral approach.^6^^–^9 Based on our prior study on transurethral cryoablation for bladder urothelial carcinoma, our team designed a novel cryoprobe which was applicable through the working channel of a regular ureteroscope (Fr-8/9.8). We conducted an animal study to investigate the safety of ureteral cryoablation in a porcine model and reported the preliminary experience of cryotherapy for patients with UTUC of a solitary kidney.

## Patients and Methods

### Liquid-Nitrogen Equipment and Cryoprobe

**Fig. 1:**
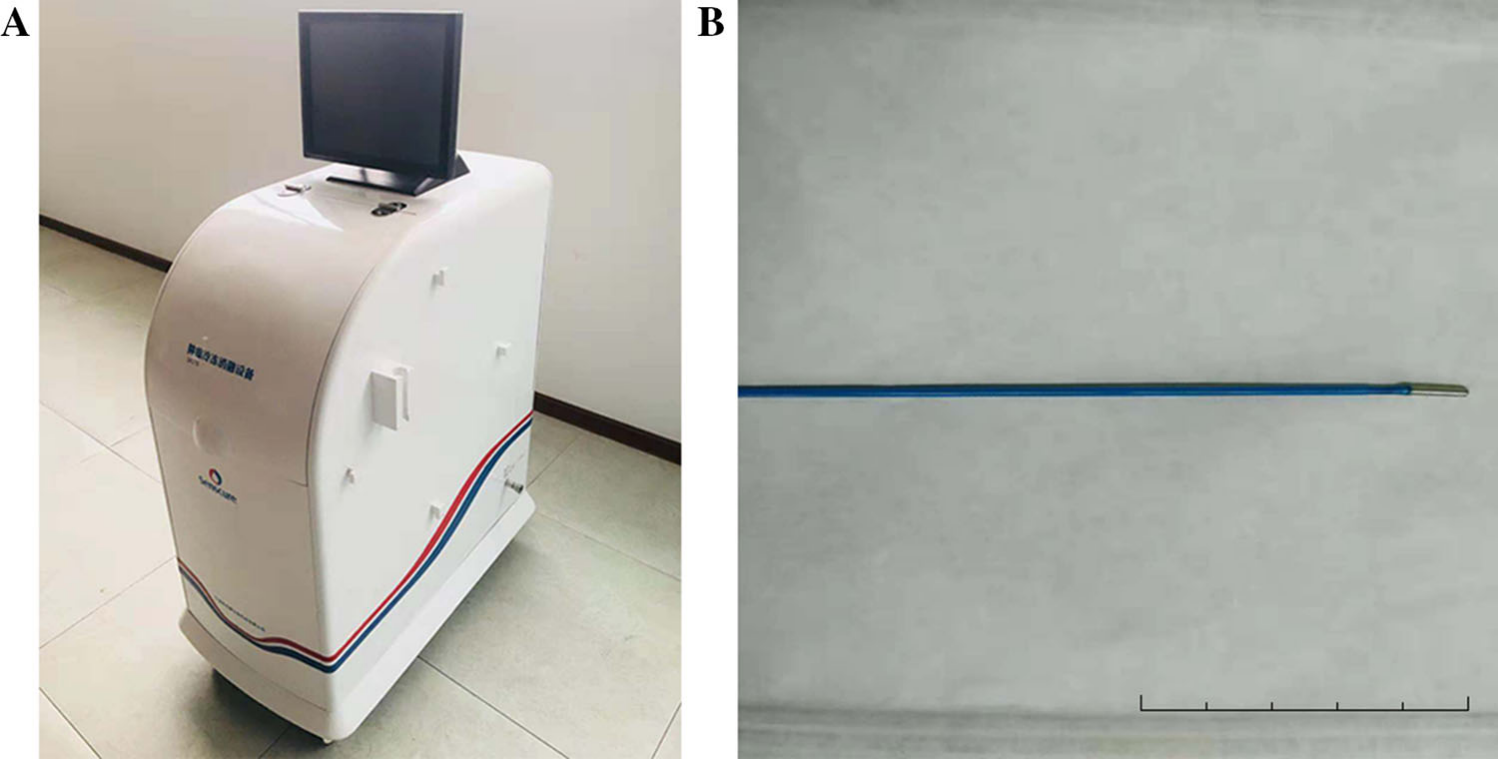
Liquid nitrogen-based cryosurgery unit: (A) control system, (B) cryoprobe

A liquid-nitrogen-based unit designed and manufactured by Senscure Company, Ningbo, Zhejiang, China was used in this study. This unit is composed of a control system and a cryoprobe (Fig. 1). The control system has a 70-L Dewar for liquid-nitrogen storage. The cryoprobe consists of an outer lumen for vacuum insulation and two inner lumens for liquid-nitrogen circulation. The cryoprobe is 99 cm in length and 1.44 mm in diameter. The working tip of the cryoprobe is 8 mm in length and 1.4 mm in diameter, being able to produce a therapeutic ice ball. The freezing duration can be controlled by regulating the liquid-nitrogen release by using the control system.

During laboratory research, a gel model experiment was conducted for measurement of the ice ball. After 2 min of freezing, the ice ball was spherical with diameter of 30 mm, as evaluated in 4% gelatin solution at 22 °C.

### Animal Study

The study was approved by the Ethics Committee of Fudan University. A total of nine white pigs (weight range 20–25 kg, age range 8–12 months) were obtained from the Experimental Teaching Center, School of Agriculture and Biology, Shanghai Jiao Tong University. All pigs were fasted for 12 h but allowed water ad libitum before surgery. Eight pigs were randomly assigned to two different groups according to the freezing duration of 60 or 90 s. The other pig was specially designed to receive a 10-min freeze.

After induction of general endotracheal anesthesia, the pigs were placed in supine position. Each pig was monitored intraoperatively by pulse oximetry, end-tidal carbon dioxide, electrocardiography, and noninvasive blood pressure. A 15-cm midline incision was made on the lower abdomen. The lower-right ureter was dissociated carefully to preserve periureteral blood supply and then transected. The cryoprobe was inserted into the proximal ureteral lumen through the ureteral stump, and the tip of the cryoprobe was placed 5–6 cm away from the stump, then cryoablation was performed according to the grouping mentioned above. During each freeze–thaw cycle, the temperature at four sites in the ureter was measured using four thermocouple thermometers. The first thermometer was bound to the tip of cryoprobe to measure the temperature of the freeze center on ureteral mucosa. The other three thermometers were fixed to corresponding ureteral serosa, 0.5 cm and 1 cm away from the freeze center, respectively. Data for the temperature change with time were recorded and reviewed. As soon as the freeze ended, warm normal saline was applied to promote ice ball thawing. After complete thawing, the ureter was carefully examined, and an Fr-4.7 double-J ureteral stent was inserted into the treated ureter. Then, ureteral reimplantation was performed. Finally, the incision was closed.

The treated ureters in each group were harvested and fixed in 10% buffered formalin at 30 min, 2 days, 4 weeks, and 3 months after cryoablation, respectively. The contralateral ureter in each pig was sampled as control. Hydronephrosis and dilation of proximal ureters were observed during the sampling procedure. Serial sections of the ureter were embedded with paraffin and stained with hematoxylin and eosin. Pathological alterations were examined to determine the acute (30 min and 2 days), subacute (4 weeks), and chronic (3 months) effects after cryoablation. For the pigs in the 3-month subgroup, the stents were removed through brief cystotomy 4 weeks after the procedure. The ureter of the ninth pig that received the longer freeze duration of 10 min was observed intraoperatively for ureteral peristalsis and urination.

Apoptotic cells were identified respectively in treated and control ureters at 2 days after cryoablation using terminal deoxynucleotidyl transferase dUTP nick end labeling (TUNEL) staining. TUNEL staining was performed using an in situ cell death detection kit (no. 11684817910; Roche Diagnostics, Shanghai, China) according to manufacturer’s instructions.

### Clinical Study

#### Patients and Inclusion

Between December 2018 and June 2020, patients referring to our center were enrolled in the study when they met the following inclusion criteria: (1) clinically suspected or pathologically confirmed urothelial carcinoma of renal pelvis or ureter, (2) no more than three lesions in unilateral upper urinary tract, (3) diameter of largest lesion less than 3 cm, (4) having a solitary kidney, and (5) strong desire for renal preservation. The exclusion criteria were: (1) clinically suspected local advanced infiltration (> cT2), lymph node (> cN0) or distant metastasis (> cM0), (2) insufficient compliance for long-term regular surveillance. Eventually, a total of six patients were enrolled in the pilot clinical study. Regular imaging and laboratory assessment were applied preoperatively.

The study protocol was reviewed and approved by the ethics committee of Huashan Hospital, Fudan University (approval no. 2019-010). The clinical trial was registered in the Chinese Clinical Trial Registry (no. ChiCTR1900026534). Written informed consent was obtained from each patient. All procedures performed in the trial involving human participants were in accordance with the ethical standards of the institutional and/or national research committee and with the 1964 Helsinki Declaration and its later amendments or comparable ethical standards.

#### Ureteroscopic Cryoablation

**Fig. 2:**
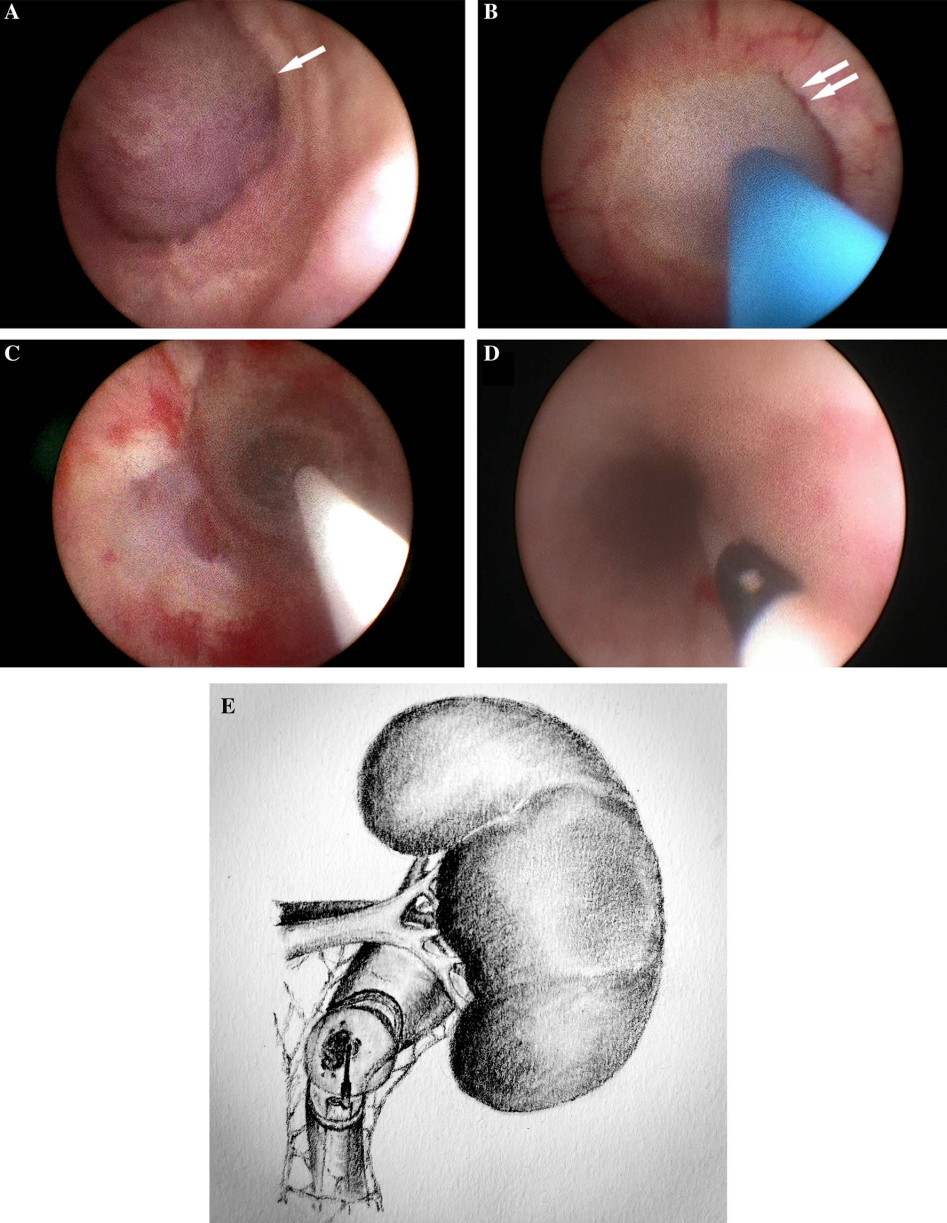
Process of ureteroscopic cryoablation for ureteral urothelial carcinoma. (A) Tumor detected, before cryoablation. Single arrow: ureteral tumor. (B) Ice ball forming. Double arrows: ice ball. (C) Ureteroscopic view after two cycles of 2-min freezing. (D) Ureteroscopic view of ureteral lumen at primary site 3 months after cryoablation. (E) Schematic diagram illustrated by the corresponding author

Patients were placed in dorsal lithotomy position under general anesthesia. An Fr-8/9.8 ureteroscope was retrogradely placed into the ureter. The renal pelvic or ureteral tumors were carefully observed, and at least three biopsy bites were obtained by forceps. The majority of the tumor was ablated with holmium:YAG laser to empty the ureteral lumen. Then, the cryoprobe was inserted through the working channel of the Fr-8/9.8 ureteroscope, and the working tip was visually placed to the area of residual tumor. The liquid-nitrogen system was then activated to form a therapeutic ice ball. Each cryoablation procedure lasted for 2 min to ensure penetration of the full-thickness ureteral wall. Thawing was accelerated by irrigation of 40 °C normal saline through the ureteroscope after each freezing process. Multiple freeze–thaw cycles (at least two cycles per site) were performed to guarantee complete freezing coverage of the tumor and its base according to its size (Fig. 2).

#### Postoperative Evaluation and Surveillance

Postoperative adverse events, such as urinary tract infection, ureteral perforation, ureteral stricture etc., were recorded according to the five-grade Clavien–Dindo classification. Postoperative serum creatinine levels during follow-up were assessed. Ureteral stents were removed under cystoscopy 6–8 weeks after cryotherapy. Computed tomography (CT) scan and ureteroscopy were scheduled every 3 months in the first year after initial cryoablation then every 6 months in the second year. Repeated cryoablation or holmium laser ablation under ureteroscope will be performed if recurrent UTUC is diagnosed during follow-up.

### Statistical Analysis

The Mann–Whitney *U*-test was used to estimate the difference between pre- and postoperative creatinine levels of all patients. Statistical significance was set at 0.05. Statistical analyses were performed with SPSS 24.0 (SPSS Inc., Chicago, IL).

## Results

### Animal Study

**Fig. 3:**
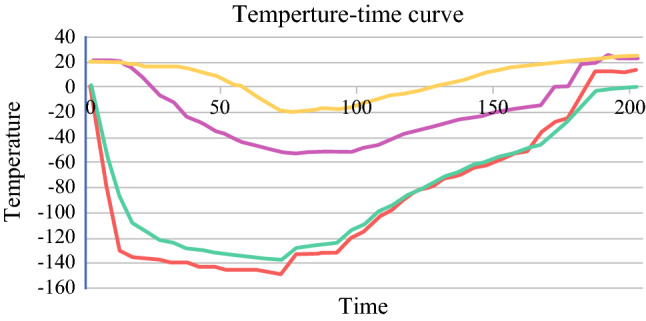
Temperature change in ureter during freezing and thawing phase (red line: ureteral mucosa, green line: ureteral serosa, purple line: 0.5 cm away from freezing center, blue line: 1 cm away from freezing center; Temperature: °C, Time: second)

All the pigs successfully tolerated the operation and restarted eating and urination within 24 h after operation as planned. During the freezing process, the temperature of frozen zone was measured to be −152 °C to −145.4 °C at the ureteral mucosa (inner surface of ureteral lumen) and −140.2 °C to −136 °C at the serosa. A therapeutic ice ball with diameter of 10–12 mm was formed in each treated ureter during the freezing cycle. Generally, the freezing process was categorized into three phases: (1) a rapid cooling phase, 0–10 s after initiation, (2) a plateau phase from 11 to 60 or 90 s, and (3) the thawing phase, after freezing (Fig. 3).

No visible hydronephrosis or ureteral dilation was detected in the animals during autopsy. In the 60-s group, neither intra- nor postoperative ureteral perforation nor stricture was observed. In the 90-s group, partial ureteral rupture occurred in one case because of inappropriate withdrawal of the cryoprobe before complete thawing, but was found recovered at the planned autopsy after 3 months. No avulsion or perforation of ureter was observed after thawing in the pig that received a 10-min freeze. Persistent peristalsis and urination from the stump of the treated ureter were detected in 5 min after complete thawing.

**Fig. 4:**
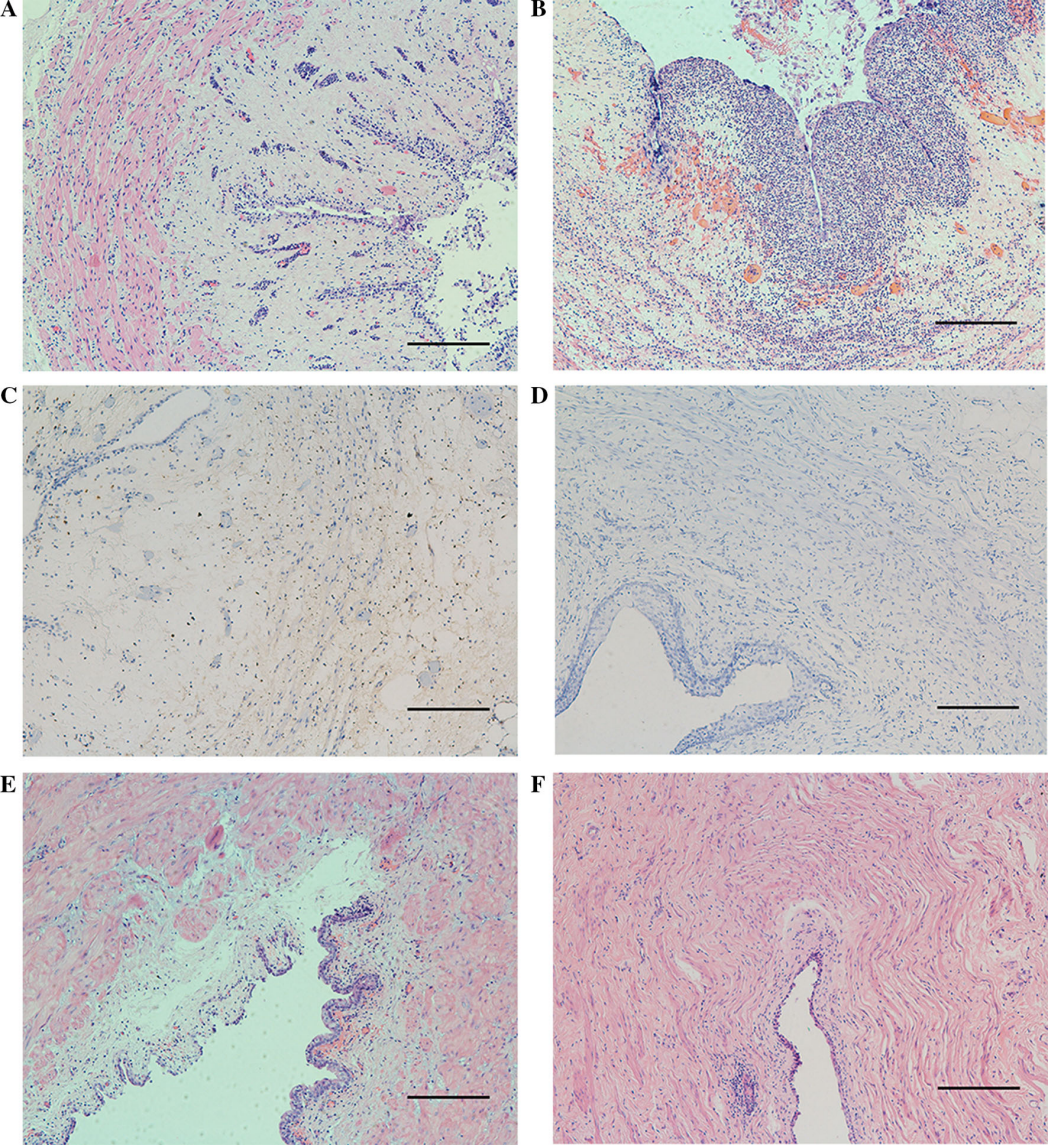
Histological changes of ureter after cryoablation (10 × 200 μm) at (A) 30 min and (B) 2 days. (C) TUNEL staining in cryoablated zone of porcine ureter at 2 days after cryoablation. (D) TUNEL staining in normal ureter. (E) At 4 weeks after cryoablation. (F) At 3 months after cryoablation. *TUNEL* terminal deoxynucleotidyl transferase dUTP nick end labeling

Histologic examination revealed the acute, subacute, and chronic alterations of ureter after cryoablation. No significant difference of histologic alterations was observed between the 60-s and 90-s group. Mucosal abscission and vessel congestion were observed in submucosa and lamina muscularis of the frozen zone at 30 min after freezing (Fig. 4A). Submucosal hemorrhage, transmural edema, infiltration of inflammatory cells, and necrosis were observed in submucosa and lamina muscularis at 2 days after freezing (Fig. 4B). Compared with the normal ureters, apoptotic cells were observed in the frozen zone of full-thickness ureteral wall (Fig. 4C, D). At the subacute stage, incomplete neoepithelialization was noted in the frozen zone, as well as fiber deposition and inflammatory infiltration. Additionally, a few hemorrhagic foci were observed in submucosa (Fig. 4E). At the chronic stage, the frozen zone showed complete reepithelialization, fiber deposition, and focal inflammatory reaction (Fig. 4F).

### Clinical Study

#### Clinical Characteristics

The six enrolled patients consisted of three males and three females. Median (range) age at diagnosis was 62.5 (49–77) years. Clinicopathological characteristics and follow-up results of all participants are presented in Table 1. Peripherally infiltrative tumor was not detected in any of the patients by preoperative CT evaluation. All patients were diagnosed UTUC of solitary kidney and received ureteroscopic cryotherapy.

**Table 1:** Clinicopathological characteristics of enrolled patients.

No.	Patient	Tumor				Cryoablation	Pathology		Follow-up		
	Gender	Age (years)	Size (cm)	Location	Number	Cycle	Stage	Grade	Duration (months)	Complication	Recurrence
1	Male	55	1.7	Renal pelvis	1	2 min * 4	N/A	LG	16	No	No
2	Female	77	0.5	Ureter	1	2 min * 2	N/A	LG	14	Ureteral stricture	No
3	Female	49	1	Ureter	3	2 min * 6	T2	HG	16	No	HG
4	Male	61	0.8	Renal pelvis	1	2 min * 4	N/A	LG	11	No	No
5	Male	64	1	Ureter	3	2 min * 6	N/A	LG	7	No	LG
6	Female	65	1.5	Renal pelvis	1	2 min * 4	N/A	LG	3	No	No

*HG* high grade, *LG* low grade.

#### Perioperative Outcomes

All the ureteroscopic procedures were performed successfully. In two patients with tumor partly located in the upper calyx, flexible ureteroscopy was applied to observe the whole lesions, and afterwards, holmium laser ablation was conducted for the remnant frozen tumor. No ureteral perforation, avulsion, or active hemorrhage was observed during the procedures. There was no significant statistical difference between pre- and postoperative serum creatinine levels (*p* = 0.82).

#### Follow-Up Results

The median (range) follow-up period was 12.5 (3–16) months. No salvage radical surgery was performed in any patients. No recurrence in situ was detected during follow-up. In one male patient with the primary tumor located in the renal pelvis, an ectopic recurrent tumor was detected and thereafter managed with repeated ablation through percutaneous nephroscopy 3 months after cryotherapy. In another, female patient, recurrent lesions were detected in the ureteral orifice and the bladder both 3 and 6 months after cryotherapy. Repeated cryotherapy was then conducted together with transurethral rection (TUR) of bladder lesions. During the follow-up, this patient presented with pain in leg and was diagnosed with bone metastasis 8 months after initial cryotherapy.

Postoperative hydronephrosis was detected in one patient at 6 months after cryotherapy. Ureteral stricture in the treated area was diagnosed in the meantime, managed with balloon dilation under ureteroscopy, and did not relapse during subsequent follow-up.

## Discussion

The efficacy of KSS for low-risk UTUC has already been recognized. Generally, endoscopic techniques are vital procedures of KSS, usually referring to tumor ablation through ureteroscopy and percutaneous access.^1^ Laser ablation remains the most dominant method among the kinds of endoscopic techniques applied so far.^1^,^10^ These therapeutic options should also be considered for selected patients, for example, those with a solitary kidney, bilateral UTUC, and renal insufficiency, even in high-risk cases. It is regrettable that patients with high-grade disease often fare poorly in consideration of existing modalities.^11^ Improving the effectiveness of tumor control while preserving renal function is thus a focus.

As mentioned above, cryotherapy has already been included in the treatment of urothelial tumor, mainly for bladder tumor so far.^5^–^9^ Compared with the bladder wall, the ureteral wall is relatively thinner. The feasibility and safety of cryoablation of ureter as a potential therapy for urothelial carcinoma remains to be explored. A recent small-sample study in a porcine model investigated the application of cryotherapy with a novel balloon cryoprobe on ureter for management of ureteral endometriosis.^12^ The liquid-nitrogen-based equipment used in the study was manufactured by the same institution as that applied in our study, while the design of the cryoprobe was different. The porcine ureter underwent intraluminal cryoablation through a small incision in the ureteral wall. Eventually, no pigs suffered postoperative complications such as perforation, abdominal adhesion, adjacent organ injury, urinomas, stricture, obstruction, or hydronephrosis. The results suggested that cryotherapy for ureter was feasible, safe, and effective, and provided us reference before cryoablation could be applied in clinical practice of managing ureteral diseases. The main difference between the cited study and our application is that our cryoprobe can be inserted through the working channel of a ureteroscope and subsequently placed to the tumor site accurately.

Before the clinical study, we performed an animal experiment to evaluate the safety of cryotherapy for ureter and obtained the appropriate parameters for freezing time. Regardless of freezing time, no perforation occurred despite full-thickness edema and necrosis in ureter tissue, suggesting that endoscopic cryoablation can maintain the integrality of the renal pelvic and ureteral lumen. Given the occurrence in the 90-s group, withdrawal of the cryoprobe after complete thawing is critical for prevention of ureteral injury. The histological alterations, including full-thickness or local inflammatory infiltration, edema, cell necrosis, and apoptosis, demonstrate that 60- and 90-s freezing were both able to induce tissue destruction in lamina muscularis. Since the thickness of porcine ureteral wall is much thinner compared with that of human ureter, longer freezing duration should be applied in human cases. These findings validate the safety and feasibility of intraluminal cryoablation in ureter.

Despite fiber deposition after cryotherapy, no ureteral stricture was observed in the animals during gross and microscopic examination. This result is in accordance with the recent study on ureteral cryoablation discussed above.^12^ Nevertheless, postoperative ureteral stricture remains a primary concern according to surgical experience. Therefore, ureteral stent indwelling after cryotherapy was recommended, as applied in the clinical study. Additionally, cryotherapy on vessel walls can result in collagen accumulation and lumen loss.^13^ In our clinical study, there was one case affected by curable ureteral stricture during the limited follow-up. It was difficult to identify the key cause of ureteral stricture as laser ablation or ureteroscopy itself may also result in this complication. Objectively speaking, evaluation of ureteral stricture still requires long-term investigation.

The short-term efficacy of cryotherapy was revealed in our clinical study. It was encouraging that no tumor recurrence was visually found at the primary tumor sites through endoscopic examination. There were two patients who had de novo lesions outside the primary sites. This may be attributed to multifocality and higher malignancy regarding high-grade tumors. As reported previously, increased risk of local recurrence is related to endoscopic management of UTUC.^2^ As is well known, residual tumor in bladder is mostly detected at the primary site after conventional resection.^14^ Unlike pathologic biopsy or re-TUR of bladder tumor, second biopsy or resection at primary sites of UTUC was infeasible considering further harm to the ureter and its potential induction of stricture. In our study, absence of recurrence in situ demonstrated the promising role of cryoablation in local tumor control to some extent. Moreover, regular surveillance was strongly recommended after cryotherapy, and repeated cryoablation was shown to be feasible for recurrence or de novo tumors.

This pilot study was not designed to be a randomized controlled study due to the low incidence of UTUC and the specific inclusion and exclusion criteria. It was performed to estimate the feasibility and safety of this novel therapeutic management for UTUC, especially for cases with a solitary kidney, and also potentially suitable for cases with bilateral tumor, renal insufficiency, inoperable by RNU, etc. Long-term follow-up is necessary to evaluate the efficacy of tumor control. As the rigid cryoprobe used in this study cannot fulfil the demand of treatment for tumors in renal calyx, a flexible cryoprobe has been under development. Despite these limitations, endoscopic cryoablation showed application prospects for management of UTUC.

In summary, the results of this study suggest that endoscopic cryotherapy is a promising technique for management of UTUC with potential benefits of improving local tumor control and renal preservation. Selected patients with UTUC of a solitary kidney, as well as those with bilateral disease or unsuitable for RNU, may benefit from cryotherapy under endoscopy regarding favorable outcome of renal function. A randomized, large-sample clinical trial is demanded to investigate the long-term efficacy and safety of this novel therapy.

## Supplementary Information

Below is the link to the electronic supplementary material.

Supplementary file1 (MP4 161721 kb)
